# An ecohealth assessment of poultry production clusters (PPCs) for the livelihood and biosecurity improvement of small poultry producers in Asia

**DOI:** 10.1186/2049-9957-4-6

**Published:** 2015-02-09

**Authors:** Libin Wang, Edi Basuno, Tuan Nguyen, Worapol Aengwanich, Nyak Ilham, Xiaoyun Li

**Affiliations:** College of Humanities and Development Studies, China Agricultural University, Qinghua Donglu 17, Beijing, 100083 China; Indonesian Center for Agriculture Socio Economic and Policy Studies, J1 Ahmad Yani 70, 16161 Bogor, Indonesia; Center for Agricultural Policy, Institute of Policy and Strategy for Agriculture and Rural Development, Hanoi, Vietnam; Faculty of Veterinary Sciences, Mahasarakham University, Maha Sarakham, 440000 Thailand

**Keywords:** Poultry production cluster (PPC), Small producers, Biosecurity, Infectious diseases, Livelihood, Ecohealth

## Abstract

**Background:**

Poultry production cluster (PPC) programs are key strategies in many Asian countries to engage small commercial poultry producers in high-value production chains and to control infectious poultry diseases. This study assessed the multiple impacts of PPCs through a transdisciplinary ecohealth approach in four Asian countries, and drew the implications for small producers to improve their livelihoods and reduce the risk of spreading infectious diseases in the poultry sector.

**Methods:**

The data collection combined both quantitative and qualitative methods. It comprised: formal structured household survey questionnaires, measuring the biosecurity level of poultry farms with a biosecurity score card; and key informant interviews. Descriptive statistics were used to process the quantitative data and a content analysis was used to process the qualitative data.

**Results:**

This research found that poultry farms in clusters do not necessarily have better economic performance than those outside PPCs. Many farmers in PPCs only consider them to be an advantage for expanding the scale of their poultry operations and improving household incomes, and they are less concerned about—and have limited capacities to—enhancing biosecurity and environmental management. We measured the biosecurity level of farms in PPCs through a 14-item checklist and found that biosecurity is generally very low across all sample sites. The increased flies, mosquitoes, rats, and smells in and around PPCs not only pollute the environment, but also cause social conflicts with the surrounding communities.

**Conclusion:**

This research concluded that a poultry cluster, mainly driven by economic objectives, is not necessarily a superior model for the control of infectious diseases. The level of biosecurity in PPCs was found to be low. Given the intensity of poultry operations in PPCs (farms are densely packed into clusters), and the close proximity to residential areas of some PPCs, the risk of spreading infectious diseases, in fact, increases. Good management and collective action for implementing biosecurity measures are key for small producers in PPCs to address common challenges and pursue health-based animal production practices.

**Electronic supplementary material:**

The online version of this article (doi:10.1186/2049-9957-4-6) contains supplementary material, which is available to authorized users.

## Multilingual abstracts

Please see Additional file 1 for translations of the abstract into the six official working languages of the United Nations.

## Background

The poultry industry in Asia is a vital source of livelihood for many of the continent’s rural communities. However, the large-scale industrial producers are squeezing out the small operators from the expanding market [1].

Since 2003, the unprecedented highly pathogenic avian influenza (HPAI) outbreaks in Asia have stalled the development of the poultry sector [2–4]. Governments in most countries have since then imposed higher animal-health standards—these are improving public health, but are also widening the gap between smallholders and large commercial producers [5–7]. The “livestock ladder”—by which smallholders climb up the scale of production and out of poverty—is missing several rungs [8]. In response, many Asian countries have promoted poultry production clusters (PPCs) as a means to drive small commercial producers into groups for higher productivity and safer poultry value chains. For example, in 2004, China initiated the “Small Livestock Raising Area” program. In 2006, Indonesia started the “Village Poultry Farming” program, Vietnam initiated the “Poultry Production Zone” program, and Thailand introduced the “Compartmentalized Poultry Farm” program. All these programs were intended for developing poultry production centers and enhancing biosecurity at the local level.

In general, a cluster is defined as a geographical concentration of interconnected companies and institutions which gain advantages through co-location [9, 10]. Cluster policies are argued to be crucial for small farmers and agribusiness, as they enable small-scale farmers to engage in higher productivity practices that are more market orientated with higher value-added production [11]. Poultry production clusters also offer more opportunities to implement biosecurity measures. The names and operations of PPCs vary in different countries, but the clusters themselves share many common characteristics. The research team in this study defined PPCs as “areas of concentrated poultry production in rural locations usually separated from residential sites, where multiple small producers practice certain economies of scale and apply standard biological safety and environmentally friendly measures, and install related facilities”.

Although there are many initiatives to develop PPCs in Asian countries, there is very limited empirical evidence on the social, economic, human health, and environmental consequences of production clusters and their implications for the control of emerging infectious diseases. Specifically lacking is an ecohealth approach to assess this production mode from a multidisciplinary perspective.

Many key issues remain debatable. It is not clear if the economic returns of producers in a PPC are better than those of traditional producers. The rapid industrialization of poultry for the wrong reasons could harm the mechanism of income generation for the poor [12]. Some studies have found that poultry density is a key risk factor for spreading infectious animal diseases [13–17].

Control of HPAI is not only a biosecurity issue, but also a challenge for development [18]. Some researchers have criticized the fact that public policy has often supported and subsidized industrial poultry production, promoting economies of scale, but ignored the equity, environmental, and health consequences [1, 19]. Is a PPC a viable option to address the equitable development issues in the poultry sector? To answer this question, a research program was jointly implemented in China, Indonesia, Vietnam, and Thailand. The main research purpose was to assess the multidimensional impacts of PPCs on small producers with specific reference to emerging infectious diseases. This paper reports the findings on the social and economic impacts of PPCs on the small producers, the impacts on the environment, as well as their effectiveness in controlling infectious diseases.

## Methods

This research applied an ecohealth approach, which perceives human health, animal health, and environmental components as an integrated system, and gathers insights from different scientific disciplines to develop sustainable solutions that transcend the health sector. Ecohealth approaches also help translate research findings into policy and action [20].

### Study sites

This study involved four Asian countries: China, Indonesia, Vietnam, and Thailand. In each country, one to three provinces with PPCs were selected (see Table 1) for field surveys based on the following criteria: a high share of poultry production in the country, existence of PPCs and intensive poultry farming, and whether its representative of the different models of PPCs in the country.

**Table 1:** **Location and number of surveyed farms, and their poultry species**

***Country***	***Location of research sites***	***Number of PPC farms surveyed and poultry species***	***Number of non-PPC farms surveyed and poultry species***
**China**	Heishan County, Liaoning Province, Northeast China	30 Layer chicken	40 Layer chicken/hens
	Xingye County, Guangxi Region, South China	40 Local broiler chicken	NA
**Indonesia**	Dawuan Sub-district, Subang District, West Java Province, Indonesia	53 Broiler chicken	31 Broiler chicken
	Sukadana Sub-district, Ciamis District, West Java Province, Indonesia	51 Male layers	NA
	Baregbeg Sub-district, Ciamis District, West Java Province, Indonesia	54 Male layers	NA
**Vietnam**	Suburban areas of Hanoi, North Vietnam	66 Mainly chicken broilers and layers	163 Mainly chicken broilers and layers
	Dong Nai Province, South Vietnam	76 Mainly chicken broilers and layers	88 Mainly chicken broilers and layers
**Thailand**	Nong Khai Province, upper northeastern region of Thailand	15 Layer chicken above the fish ponds	15 Layer chicken above the fish ponds
	Nakhon Phanom Province, upper northeastern region of Thailand	15 Layer chicken	15 Layer chicken
	Mahasarakham Province, center of the northeastern region of Thailand	15 Chicken broilers and layers	15 Chicken broilers and layers

### Sampling and data collection

This research intended to make a systematic comparison between PPC and non-PPC producers in the four countries. However, since there were limited numbers of non-PPC poultry farms in some of the research locations, these samples were not sufficient for analysis (Xingye County, China; Sukadana and Baregbeg sub-district, Indonesia). In addition, the PPCs in each country have different social and economic contexts, natural resource endowments, management modes, and poultry species, which constrained the use of uniform sampling procedures across the research sites.

In each country, the data collection combined both quantitative and qualitative methods:

1. Formal structured household survey questionnaires were used to collect quantitative information on the social and economic situation of small-scale poultry farmers: farmer household profiles, poultry species and scales of operation, poultry management mode, costs, and benefits of poultry raising. Two targeted groups were selected: small producers located inside the PPCs, and typical small producers located locally, but outside of the PPCs. The information on the location and numbers of PPC and non-PPC farms were acquired from local government agencies and village committees. The selection of PPCs was based upon the representativeness of the PPCs in the local area and the ease of access to the site for the researchers. From the selected PPCs, the producers were selected by random sampling based on the sample size needs, and taking time and financial constraints into account. The non-PPC farms were selected from the farms that were located in the same village or nearby to the PPC farms, and had similar poultry species and operational scales as them. Table 1 summarizes the sample information.
2. Altogether, 10–15 key informant interviews were conducted in each research site. These interviews were mainly conducted with the representative poultry farmers, village committee members, village health and veterinary workers, representatives from the contract companies, and government officials. Semi-structured interview guides were used to collect the respondent perceptions and concerns relating to the PPCs.
3. A biosecurity score card was developed to meaures the level of application of biosecurity measures in the surveyed farms. This score card, in accordance with the Food and Agriculture Organization of the United Nation’s (FAO’s) concept of biosecurity, was developed by a professional consultant. There are 14 indicators in total, which include different control measures: (1) Attractiveness to wild birds; (2) Wild bird protection; (3) Measures related to staff on the farm; (4) Measures for incoming poultry; (5) Measures for visitors; (6) Measures for traders; (7) Measures for equipment and vehicles; (8) Source and treatment of water; (9) Source of feed; (10) Local environment: distance from the road and other farms; (11) Types of poultry on the farm; (12) Capacity to clean and disinfect the farm; (13) Measures taken at the entrance to poultry sheds; and (14) Availability of biosecurity plans. For each indicator, a grading scale was established of 0, 1, 2, or 3, with zero as the lowest score (poor condition) and 3 as the highest level of security measure, totaling a possible score of 42. The main method for the scoring consisted of observations during farm visits and questions posed to farm owners/managers. The data were collected from sample farms in PPCs and non-PPCs whenever possible, and the samples were the same as those in the formal structured household interviews, as indicated in Table 1.

The four country teams (researchers together with trained research assistants) collected data from January to December in 2012.

### Data analysis

The data from the quantitative surveys and the biosecurity scores were entered into the SPSS software package. Descriptive statistics were used to compare the differences in terms of economic and biosecurity aspects between PPC farms and non-PPC farms. Content analysis was used to analyze information collected from key informant interviews. The research teams analyzed the presence, meanings, and relationships of qualitative information, and then made inferences about the findings and their meanings in a local context. The onsite observation results, collected through field visits, were documented and the key issues were identified, described, and discussed.

## Results

### Location, structure, and management of PPCs

The first observation regarding the PPCs derives from a geographical description of the location—that is, location differences between PPCs and non-PPCs, and between PPCs and the village communities nearby, as well as differences across countries. In general, a number of small-scale commercial poultry farms that are located near each other form a PPC. In comparison, non-PPC farms are located more sparsely in and around villages. The number of farms within surveyed PPCs varied in different countries, from five to 60, and the distance from one farm to the next in the same cluster varied from a couple of meters to about 100 meters.

The surveyed clusters in the four countries are located from 500–3,000 meters away from the residential areas, or in the residential areas, depending on the history of the PPCs and the availability of land (see Table 2). For example, the two PPCs in Sukadana and Baregbeg, Indonesia, are located in residential areas, and the distance from the poultry pens to peoples’ houses is less than 20 meters for about 60% of the 105 farms in these two PPCs.

**Table 2:** **Sites, number of farms, management modes, poultry scale, and net income in surveyed PPCs**

***Country***	***PPCs***	***Distance from residential areas (RA)***	***Number of farms in each PPC***	***Management modes in PPCs***	***Average poultry size/cycle/farm in PPCs (heads) cycles/year***	***Poultry size/cycle/farm in non-PPCs (heads) cycles/year***	***Annual net income from poultry/farm in PPCs in 2011 (USD)***	***Annual net income from poultry/farm in non-PPCs in 2011 (USD)***
**China**	Heishan	300–500 m	10–20	Independent	5,532 Layers 12–14 months/cycle	3,395 Layers 12–14 months/cycle	16,987	8,805
	Xingye	500–700 m	10–30	Contract farming	6,725 Local broilers 2–3 cycles	-	8,135	-
**Indonesia**	Dawuan	2.8 km	53	Contract farming	5,138 Broiler 5–6 cycles	4,577 Broiler 6 cycles	2,266	3,537
	Sukadana	In RA	51	Contract farming	2,854 Male layer 4–5 cycles	-	655	-
	Baregbeg	In RA	54	Contract farming	2,260 Male layer 3–4 cycles	-	496	
**Vietnam**	Hanoi	500–700 m	5–20	Mostly contract farming	5,354 Broiler 3–4 cycles	5,267 Broiler	16,522	12,931
	Hanoi	500–700 m	5–20	Mostly contract farming	4,316 Layers 1–2 cycles	4,233 Layers	12,260	19,588
	Dong Nai	In RA	7–20	Mostly contract farming	5,651 Broliers 3–4 cycles	8,478 Broilers	15,278	30,268
	Dong Nai	In RA	7–20	Mostly contract farming	25,460 Layers 1-2 cycles	16,817 Layers	114,033	61,734
**Thailand**	Nong Khai	1–1.5 km away from RA	30–50	Independent	2,035 Layers 12–18 months/cycle	1,802 Layers	25,093	20,515
	Nakhon Phanom	In RA	Around 50	Cooperative	4,932 Layers 12–18 months/cycle	5,510 Layers	40,026	44,333
	Maha Sarakham	1–1.5 km away from RA	50–60	Contract farming	23,893 Broilers 3–4 cycles	21,026 Broilers	16,287	13,333

*Source*: Field survey results.

*Note:* (i) the production cycle for layers is from the time of producing eggs to the time of retirement.

(ii) RA-residential areas, m-meters, km-kilo meters.

(iii) the currency exchange rate was based upon the exchange rate of the main banks in each research country during the time of the fieldwork in 2012.

The field surveys found that the “cluster” is the main form of structure of small commercial producers. The reason is that by locating close together, small farms can reduce the transaction costs because the input suppliers and dealers are more willing to come to serve these small farmers which are close to each other, and the companies are more interested in contracting the farms in clusters to reduce the costs of management, transportation, etc. In many research sites, such as those in Thailand and Indonesia, it is difficult to find scattered, individual poultry farms outside PPCs. However, there is no official data available on how many PPCs there are in each site, since the cluster is not a formal unit for survey and analysis in governmental statistical data.

This research found that PPCs have developed both as a result of government policy and as spontaneous events, and some as a mixture of both. Basically, there are three main forces that form PPCs in the four studied countries.

The first is government policies and programs to support small commercial producers to build up PPCs outside of residential areas, with the following rationales:

1. It would reach economies of scale in terms of production at the cluster level, so that the farmers can be more competitive in the market.
2. It would reduce the possibilities of spreading diseases from animals to humans.
3. It would be easier and fairer to ask the producers to follow the same procedures and requirements on biosecurity and environment management, and be more cost-effective to install related facilities (such as treatment of waste).

The second is the large commercial companies that supply inputs and purchase outputs. They select a number of contract farmers in a concentrated area so that they can supervise and manage them more efficiently. For example, in Dawuan PPC, Indonesia, three poultry farmers established a partnership with a company in 1989. Over 20 years, 55 nearby farmers joined the contract group and formed a big cluster.

The third is farmers’ social networks, consisting of extended families, relatives, neighbors, friends, or cooperatives who build poultry farms near each other to facilitate mutual social support and connections, and to learn from each other. For example, one successful poultry farmer in Nong Khai, Thailand helped his family members and relatives move in and build poultry farms near him since the 1990s, which eventually formed a PPC with 50 farms.

Three management modes were observed in the surveyed PPCs: (i) independent, which means that the producers in the PPCs are independent of each other for their production activities and lack of collective action in PPCs; (ii) cooperatives (the producers build up a cooperative to coordinate some of the collective action such as the procurement of inputs); and (iii) contract farming (the producers are under contract farming arrangements with big companies). Usually, the companies provide production inputs and technical services and buy the harvested chicken at pre-fixed prices.

### Economic performance

The average poultry scale per production cycle per farm and the species in PPCs and non-PPCs differ in different countries (see Table 2). The range is from 2,000–7,000 birds per cycle for the majority of the surveyed farms. Chicken layers and broilers are the main types. If we only look at the small farmers under 10,000 birds per cycle, the farmers in Indonesia obtained the lowest annual net income from poultry, ranging from USD 496 to 2,266. Farmers in Thailand earned the highest, ranging from USD 25,093 to 40,026. China and Vietnam were in the middle, ranging from USD 8,135 to 30,268.

Although the considerable variation in poultry production made it difficult to make systematic comparisons, the following points can be made (which were also supported by the interviews and onsite observations):

1. The poultry scale in PPCs can be similar, smaller, or larger than that of their counterpart farms outside of the PPCs. Likewise, the income of the PPC farmers from production could be better, lower, or similar to their non-PPC counterparts (see Table 2).
2. Given the similar poultry species and operation scales, the PPC farmers did not necessarily make higher profits, since the producers outside the PPCs may have higher technical and management skills and achieve comparable economic performances.
3. The management mode of poultry production could also make a difference. If the producers in PPCs are under contract farming, the companies will take part of the profits, which thus reduces the economic returns to the producers. In comparison, the counterpart producers outside the PPCs could have more economic gains by operating independently.

However, by locating together, the farmers in PPCs are more likely to engage in contract farming with large companies. For example, in Hanoi, Vietnam, 69% of the surveyed farms in PPCs are under contract farming arrangements, while only 36% of surveyed farms outside PPCs are contracted. In Indonesia, all three surveyed PPCs (each with 50–60 farms) are under the contract farming system. Contract farming has helped small commercial producers to cope with the financial, technical, and market barriers to operate their poultry businesses, and brought them a more reliable, though not necessarily higher, income than that of the non-PPC producers.

### Social and environment impacts

In all four countries, it was observed that income from poultry production was used to build new houses and renovate old houses, buy new vehicles, and support children’s education. The meat and eggs from the available poultry also improved the nutrition status of most families. Women also played active roles in PPCs since their husbands alone cannot do all the work given the commercial scale of a poultry operation. In Baregbeg PPC, Indonesia, women mainly operate the PPC farms as the men migrate out of the district for non-farm jobs. The income from poultry contributed to 47% of the household income.

In comparison with poultry farmers outside PPCs, the farmers within PPCs have more frequent social exchanges, due to their close proximity, such as exchanging information related to markets and disease control. However, some social exchanges may bring the risk of infectious diseases. For example, it is common for PPC farmers to exchange labor, such as helping each other apply vaccinations, remove poultry manure, and catch chickens during harvest. One benefit of doing this is saving costs of hired labor. In Dawuan PPC, Indonesia, 90% of PPC farmers and 55% of non-PPC farmers exchanged labor.

The PPC farms do not show better results in terms of environmental protection. Most of the farms inside PPCs in the four countries, as their counterparts in non-PPCs, do not have wastewater and manure management systems. For example, in Vietnam, about 64% of the surveyed PPC farms and 66% of the non-PPC farms let wastewater go directly into the ground or to nearby ponds, rivers, or streams. In China, the farmers in PPCs in Heishan just let the wastewater go along the canals/ditches used for drainage of rain water and simply leave the poultry manure outside the poultry pens before the manure is sold. When it rains, the manure and the wastewater will flow freely over the PPC land and into nearby farm fields.

It is evident from onsite observations and key informant interviews that there are higher concentrations of flies, mosquitoes, rats, and various smells in and around PPCs, especially during rainy seasons. These not only increase the risk of disease transmission to animals and humans, but also disturb community neighbors and sometimes cause serious social conflicts. For example, the Dawuan PPC (with 55 farms) in Indonesia was originally located in a residential area and later moved to a paddy field two to three kilometers away because of complaints from community members. However, the smells and flies still spread to the communities at harvest time when transportation vehicles pass through the residential areas, causing a protest on the presence of PPCs by 500 people in 2010.

To reduce complains from the community neighbors, PPC farmers donated money to community affairs and provided chickens for religious events, gave several chickens as gifts to their neighbors during harvest time, gave away poultry manure to neighbors for free, and hired laborers from the communities for temporary jobs. In these ways, the PPC farmers attempted to keep harmony with the surrounding community.

### Animal and human health

Overall, the biosecurity scores for poultry farms in PPCs were not high (see Table 3). The scores were around 20 out of 42 in most of the research sites. In the four countries, the farms in PPCs are generally weak on the following indicators: the distance from other farms; biosecurity precautions for visitors, traders, equipment, and vehicles; control measures at the entrance to poultry sheds; measures for incoming poultry; the capacities to clean and disinfect the farm; and protection measures for poultry workers.

**Table 3:** **Biosecurity scores for farms in research sites**

***Country***	***PPCs***	***Management modes in PPCs***	***Score for PPC farms***	***Scores for non-PPC farms***
**China**	Heishan	Independent	22.86	19.11
	Xingye	Contract farming	19.22	-
**Indonesia**	Dawuan	Contract farming	18.00	-
	Sukadana	Contract farming	9.97	-
	Baregbeg	Contract farming	7.40	-
**Vietnam**	Hanoi and Dong Nai	Mostly contract farming	22.20	21.70
**Thailand**	Nong Khai	Independent	17.22	17.64
	Nakhon Phanom	Cooperative	24.08	24.36
	Maha Sarakham	Contract farming	34.86	37.10

*Source:* Biosecurity scoring under this research; out of a possible total score of 42.

The high density of poultry farms in PPCs means a low score for the indicator “distance from other farms”. The two PPCs located in residential areas in Indonesia got “0” on this indicator, as most of the farms are only about 10–25 meters away from the next farm. This close proximity facilitates the spreading of diseases. The layer farms in PPCs in Heishan County, China are just a couple of meters away from each other, and the farmers complained that if the poultry in one farm gets sick, the disease could spread to all the other farms within three to five days.

Most farms in PPCs do not have fences, gates, or barriers. Poultry transport vehicles are allowed on farms without cleaning or disinfection. Most farms do not have special control measures for visitors and traders, such as providing special clothes or footwear.

In general, the farmers in PPCs, like the non-PPC farmers, underestimate the risks of poultry-related disease transmission to humans. Most of the farmers do not always wear protective gloves, masks, shoes, or clothes as they also carry out other work on the farm and feel it is inconvenient to keep changing.

Farmers in PPCs also reported on respiratory problems associated with poultry production. For example, about 50% of farmers from PPCs in China reported that they have respiratory problems associated with poultry production, especially with the dust from feed preparation, the smells from removing the manure and spraying disinfectant liquids, and the hot and humid atmosphere in the brooder houses.

The private sector appears to have played an important role in enhancing biosecurity measures in PPCs with contract farms. The overall observation in the four countries is that the PPCs under contract farming arrangements have higher biosecurity measures than the PPCs consisting of independent farms. The main reason is that the contract companies have set standard procedures for biosecurity, and also assigned technical staff to supervise and guide farmers’ practices on disease control on a regular basis. For example, the contract companies in Xingye, China collected poultry samples from contract farms for blood testing to check for antibody every 45 days. In these PPCs, it is easier for different producers to follow the same procedures and standards, such as the “all in and all out” measures for poultry flocks.

On the other hand, in the PPCs consisting of independent farms, the producers usually cannot take collective action to follow the same procedures and standards. For example, they often do not implement “all in and all out,” as the producers have different forecasts on market prospects and different priorities in their personal lives, as well as different production schedules. For example, in China, the independent farmers in Heishan raise different animals in PPCs based on market demand.

There is only one cooperative PPC identified. It is in the Nakhon Phanom Province, Thailand, with a biosecurity score of 24.08, higher than the independent PPC in Nong Khai (17.22) and lower than the contract farming PPC in Maha Sarakham (34.86).

Overall, the field surveys showed that governments tended to play limited roles in supervising biosecurity and human health issues. For example, there is a lack of technical services provided by governments for small poultry farmers. Governments tend to think that poultry farm operation is a private sector issue and consequently leave the farmers and the companies to take the main responsibilities for biosecurity.

## Discussion

Although there is a wealth of research and initiatives relating to clusters in general, remarkably little attention has been paid to clusters in developing countries, and even less information is available on agricultural clusters [11]. This study attempted to capture a more complete picture of PPCs in four Asian countries and draw some implications for epidemiology. Although constrained by the divergence of conditions in each country, a number of important observations can be made regarding the emergence of PPCs in the Asian region.

First, we observe that, in general, the cluster is the main form of production for small commercial poultry producers as it enables them to survive and compete with large commercial companies. However, this study shows that the poultry farms in clusters do not necessarily have better economic performances than those outside PPCs, though the existence of PPCs does give farmers a better chance of engaging in contract farming. It enables them to cope with the technical, health, and market barriers of poultry production, and have a more reliable, though not necessarily higher, income. But it is also evident that entrepreneurial non-PPC farmers can reach these goals as well. One of the reasons for this is that companies will take a big share of the profits away and thus reduce the income of the contract farmers in PPCs.

Poultry production clusters were not only driven by government policies and private sector trends, but also by farmers’ social networks. This study found that there is active social exchange and interaction within PPCs (such as the exchange of labor), and between PPCs and surrounding communities (such as giving manure and poultry to neighbors as gifts). These enhanced social exchange activities take advantage of the proximity in cluster settings, but also pose a higher risk of spreading diseases. For example, moving labor between different farms may spread diseases from one farm to another.

The research also characterized a discrepancy between government policy intentions and farmers’ priorities. Governments expected to use the PPCs as a model to reach the scale of economies and also enhance farm biosecurity through standardizing disease control measures in the PPCs. However, many poultry farmers only considered PPCs as an advantage to expand the scale of their poultry operations and improve their household incomes. Farmers were less concerned about biosecurity and environmental management.

The level of biosecurity in PPCs was found to be low due to socioeconomic factors and poor incentives for farmers to apply strict biosecurity measures. The high impact of a disease does not guarantee high benefits from its control [21]. Given the intensity of poultry operations in PPCs (farms are densely packed into clusters), and the close proximity to residential areas in some PPCs, the risk of spreading transmissible diseases increases.

Poultry production clusters also pose negative impacts on the environment. Most of the farms do not install equipment to process wastewater and poultry manure, which pollutes the environment, especially during rainy seasons. It is evident that the number of flies, mosquitoes, rats, and smells increase in PPCs, which not only disturbs the local people, but also increases the risk of disease transmission. The poultry farmers in PPCs also underestimate the risk of disease transmission between poultry and humans, as they do not always take protective measures. This may increase their exposure to risks with the intensity of poultry farming in clusters.

Governments tended to play a weak role in supervising and enhancing the biosecurity measures in PPCs. There is a lack of technical services and regular supervision provided to small farmers in PPCs by the local livestock officials. Government roles could be greatly enhanced to improve the biosecurity status of farm units and thereby improve the livelihoods of farmers in PPCs.

This research demonstrated that the cluster model by itself is not a superior model for disease control compared to the traditional poultry production mode, unless the producers in PPCs take concerted collective action for biosecurity measures. Therefore, collective action is the milestone for clusters [11], which is also the key to reducing the potential transmission of animal diseases. Under contract farming, PPCs take better biosecurity measures than independent producers as private companies can instruct farmers to take collective action for disease control. In this regard, the private sector plays an important role for disease control in clusters.

Increasingly, agricultural cluster initiatives are seen as a key approach to help promote the agricultural sector of developing countries [11]. The results of this study have made it possible to identify potential interventions for control of animal diseases and for improving the livelihoods of small farmers in the clusters.

Governments can place more emphasis on using the “cluster” as a unit of surveillance and intervention, as it is the main form of existence of small commercial producers in the Asian countries surveyed. Therefore, governments can include the clusters in their regular supervision and monitoring activities for animal disease control. The cluster initiatives should emphasize on organizational development of the producers in PPCs, and strengthen the linkage between the clusters and the private sector in the high production value chain. For example, governments can help small producers in the clusters set up farmers’ cooperatives, and provide support and seed funding for the operation of these cooperatives. Governments can also help farmers contact poultry companies so that the farmers and companies can make contract arrangements. Governments should also regulate companies’ behaviors, and urge them to enhance the environment management in PPC areas. Governments should also monitor the environment status in PPC areas regularly. Governments can also consider improving public services for farmers to enhance their technical capacities and increase their economic benefits, thus increasing their incentives for disease control. For example, governments can provide technical training and services to farmers; provide the farmers with information on the market; issue farmers certificates for biosecure farms and safe products; and develop and introduce low cost biosecurity and environment protection measures which suit farmers’ capacities and interests. Government awareness programs should also target the social exchange activities that may generate risks.

The private sector should enhance the technical training of small producers and increase the awareness of small producers on biosecurity in poultry production. Companies should also take measures to reduce the negative impacts on the environment which will thus reduce social conflicts with the communities surrounding the PPCs.

## Conclusion

The PPC model, which concentrates on different poultry farms in one area, is mainly driven by economic motives to achieve a new scale of economy and reduce transaction costs, however, it contradicts biosecurity requirements to prevent disease transmission by reducing poultry densities and isolating farms. Though PPCs are crucial for small producers to stay in the poultry business and maintain their livelihoods, small producers are more interested in expanding their poultry business and generating more income through PPCs, and are less concerned about enhancing biosecurity and environmental management. Given the intensity of poultry operations in PPCs (farms are densely packed into clusters) and the close proximity to residential areas in some PPCs, the risk of spreading infectious diseases increases. This is a paradox that can at least be partly resolved with good support services—from both the private and public sector—to ensure that farmers in all arrangements are better able to improve their livelihoods, while exercising good practices in biosecurity and environmental control. This research shows that good management and collective action in PPCs are the keys to reducing biosecurity risks.

### Statement on ethical clearance

Each research team involved in this study received ethical clearance from the related authorities in each country before the study began.

## Electronic supplementary material

Additional file 1: **Multilingual abstracts in the six official working languages of the United Nations.** (PDF 229 KB)
