# Supplementary material for: An ecohealth assessment of poultry production clusters (PPCs) for the livelihood and biosecurity improvement of small poultry producers in Asia
Source: Infect Dis Poverty. 2015 Feb 9;4:6. doi: 10.1186/2049-9957-4-6 (PMC4429460; doi:10.1186/2049-9957-4-6)
Supplement: Supplementary file 1 — Additional file 1: Multilingual abstracts in the six official working languages of the United Nations. (PDF 229 KB) [file 40249_2014_98_MOESM1_ESM.pdf]

Translation of the abstract into the six official working languages of the United Nations

تقييم الصحة البيئية "Ecohealth" في تجمعات مزارع الدواجن لتعزيز الأمن الحيوي والدخل في المزارع الصغيرة لإنتاج الدواجن في آسيا

ليبني وانغ، إيدي باسونو، توان نجوين، وورابول إنجوانيش، نياك إلهام، شياويون لي

الملخص

الخلفية: تشكل البرامج التي تستهدف تجمعات مزارع الدواجن جزءاً أساسياً من استراتيجيات العديد من دول آسيا، وتهدف إلى إشراك المزارع الصغيرة لإنتاج الدواجن في سلسلة الإنتاج عالي القيمة، إلى جانب حثها على الحد من انتشار أمراض الدواجن المعدية. وسعت هذه الدراسة إلى تقييم الآثار المتعددة الجوانب لإنتاج الدواجن بالاعتماد على مناهج الصحة البيئية "Ecohealth" في أربع دول آسيوية، ومشاركة هذه الآثار مع المزارع الصغيرة لإنتاج الدواجن لتحسين مستوى دخلها والحد من خطورة انتشار الأمراض المعدية في قطاع الدواجن.

المناهج: تضمت عملية جمع البيانات مناهج نوعية وكمية، حيث شملت استبيانات مسح منزلي منظمة، وقياس مستوى الأمن الحيوي لمزارع الدواجن من خلال بطاقة لتسجيل نقاط الأمن الحيوي، وإجراء مقابلات مع مخبرين رئيسين. كما تم استخدام إحصائيات وصفية لإنتاج البيانات الكمية، بينما تم تحليل المحتوى لإنتاج البيانات النوعية.

النتائج: كشفت الدراسة عن أن الأداء الاقتصادي لتجمعات مزارع الدواجن لا يكون بالضرورة أفضل بالمقارنة مع المزارع التي تكون خارج نطاق هذه التجمعات. ويرى العديد من المزارعين في هذه التجمعات أن ما يميزها هو إمكانية توسيع نطاق عملياتهم وتحسين دخلهم، إلا أنهم غير مهتمين بتعزيز الأمن الحيوي وإدارة البيئة، عدا عن أنهم لا يملكون السبل الكافية لتحقيق ذلك. وقد تم قياس مستوى الأمن الحيوي في تجمعات مزارع الدواجن بالاعتماد على قائمة تدقيق تضم 14 نقطة، ووجدنا عموماً بأن الأمن الحيوي منخفض جداً في كافة المواقع التي شملتها الدراسة. ولا يقتصر الأثر السلبي لانتشار الذباب والباعوض والجرذان والروائح الكريهة داخل وحول تجمعات المزارع على تلوث البيئة وحسب، بل يساهم كذلك في خلق مشاكل اجتماعية مع الأهالي في المجتمعات المحيطة.

الخلاصة: كشف البحث عن أن تجمعات مزارع الدواجن التي يعود السبب في إنشائها إلى أهداف اقتصادية بحته، لا تعد نموذج أفضل للحد من انتشار الأمراض المعدية، حيث أن مستوى الأمن الحيوي في هذه التجمعات منخفض جداً. وبسبب كثافة العمليات في هذه التجمعات، حيث أنها مكتظة بالمزارع، إلى جانب قرب بعضها من المناطق السكنية، تصبح الخطورة في انتشار الأمراض المعدية أعلى. وفي ضوء ذلك، تعد الإدارة الجيدة والعمل الجماعي لتنفيذ إجراءات الأمن الحيوي من الأمور الأساسية للمزارع الصغيرة في هذه التجمعات للتعامل مع التحديات والسعي نحو تبني إجراءات الإنتاج الحيواني التي تأخذ بعين الاعتبار الجوانب الصحية.

Translated from English version into Arabic by noor86, through

用生态健康视角评价家禽养殖小区对于亚洲小规模养殖户生计和生物安全改善的影响

Li-bin Wang, Edi Basuno, Tuan Nguyen, Worapol Aengwanich, Nyak Ilham, Xiaoyun Li

摘要：

引言：对于许多亚洲国家来说，家禽养殖小区是把小规模家禽养殖户纳入高价值链和控制家禽传染病的重要措施。本研究采用跨学科的生态健康方法，在四个亚洲国家评价了养殖小区的多重影响，并分析了研究结果对于在家禽行业改善小型养殖户生计和降低传染病风险的含义。

方法：数据搜集采用了定性和定量的方法，包括：正式的结构化家庭调查问卷，测量养鸡场生物安全水平的生物安全评分卡，

以及关键信息人访谈。描述性统计用于处理定量信息，内容分析法用于处理定性信息。
结果：研究结果表明，小区内的养殖场比起小区外的养殖场，并不一定具有更好的经济表现。小区内的许多养殖户仅仅是把小区当成扩大养殖规模和提高家庭收入的一个有利条件，他们不太关心、也不具备充分的能力来加强小区内的生物安全和环境管理。我们用一个 14 项的评分卡来衡量小区内的生物安全水平，发现所有样本养殖场的生物安全水平都比较低，在养殖小区内部和附近增加的苍蝇、蚊子、老鼠和难闻的味道，不仅污染了环境，而且还引起了与周围社区的社会冲突。
结论：本研究认为，主要由经济目标驱使的养殖小区，对于控制传染病来说并不是一个更好的养殖模式。养殖小区内的生物安全水平偏低。由于小区内养殖密度较高（鸡舍之间紧紧相邻），而且有的养殖小区离居民居住区非常近，因此传播传染病的风险实际上是增加了。良好的管理和执行生物安全措施方面的集体行动是小区内农户应对共同的挑战和开展健康的家禽生产活动的关键。

Translated from English version into Chinese by Li-bin Wang, through

La santé des écosystèmes en zone de production avicole et l'amélioration des moyens de subsistance et de la biosécurité des petits producteurs avicoles en Asie

Libin Wang, Edi Basuno, Tuan Nguyen, Worapol Aengwanich, Nyak Ilham, Xiaoyun Li

Résumé

Contexte : Dans de nombreux pays d'Asie, les programmes de création de zones de production avicole sont indispensables pour permettre aux petits producteurs avicoles d'intégrer les grandes chaînes de production et pour empêcher la propagation des maladies avicoles infectieuses. La présente étude consiste en une évaluation transdisciplinaire des répercussions de la création de zones de production avicoles sur la santé des écosystèmes dans quatre pays d'Asie. Elle mène à la conclusion que les petits producteurs devraient améliorer leurs moyens de subsistance et réduire les risques de propagation de maladies infectieuses dans l'industrie avicole.
Méthodes : Les données ont été recueillies à l'aide de méthodes quantitatives et qualitatives. Elles comprennent : des questionnaires adressés aux ménages, servant à mesurer la biosécurité des fermes avicoles en fonction d'une échelle, ainsi que des entrevues avec des intervenants clés. On a eu recours à la statistique descriptive pour le traitement des données quantitatives et à l'analyse du contenu dans le cas des données qualitatives.
Résultats : La présente étude a révélé que le regroupement des fermes avicoles ne se traduit pas nécessairement par un rendement économique supérieur à ce qui s'observe à l'extérieur de ces zones. Beaucoup de producteurs qui se trouvent en zone de production avicole trouvent que celles-ci sont un bon moyen d'élargir leurs opérations et d'améliorer le revenu de leur ménage, et ils se préoccupent relativement peu de l'amélioration de la biosécurité et de la gestion environnementale, leur capacité à le faire étant limitée. Nous avons mesuré la biosécurité de fermes en zone de production avicole à partir de 14 critères et avons découvert qu'elle est généralement très faible dans tous les échantillons observés. L'abondance de mouches, de moustiques, de rats et d'odeurs dans les zones de production avicole et autour d'elles pollue non seulement l'environnement, mais sont aussi source de conflits sociaux avec les communautés avoisinantes.
Conclusions : La présente étude conclut que les zones de production avicole, dont les principaux objectifs sont économiques, ne constituent pas nécessairement un modèle supérieur en ce qui a trait à la prévention de la propagation des maladies infectieuses. Le degré de biosécurité observé dans les zones de production avicole était faible. Vu l'intensité des activités d'élevage avicole dans ces zones (les fermes y sont hautement concentrées) et l'étroite proximité de certaines d'entre elles avec des secteurs résidentiels, le risque de propagation de maladies infectieuses se trouve en effet accru. La bonne gestion et l'application collective des mesures de biosécurité sont essentielles à la résolution des problèmes courants et à l'exercice de méthodes d'élevage sanitaires dans les zones de production avicole.

Translated from English version into French by OlivierBilodeau, through

TRANSLATORS
WITHOUT BORDERS

Оценка экоздоровья птицеводческих производственных кластеров (ППК) в целях улучшения средств к существованию и биобезопасности малых производителей птицы в Азии

Либинь Ван, Эди Басуно, Туан Нгуен, Ворапол Аенгванич, Ниак Илхам, Сяоюнь Ли

Аннотация

Предпосылки: Программы птицеводческих производственных кластеров (ППК) во многих азиатских странах являются ключевыми стратегиями по вовлечению малых коммерческих производителей птицы в цепи производства высокого уровня и для контроля инфекционных заболеваний птиц. В настоящем исследовании оценены многочисленные воздействия ППК путем междисциплинарного подхода к экоздоровью в четырех азиатских странах и очерчены их последствия для малых производителей в плане улучшения их средств к существованию и снижения риска распространения инфекционных заболеваний в птицеводческом секторе.

Методы: Сбор данных производился на основе как количественных, так и качественных методов. К ним относились: опросники домохозяйств с формальной структурой, замеры уровня биобезопасности птицеводческих ферм с помощью таблицы оценки биобезопасности и опросы ключевых информантов. Для обработки количественных данных использовалась описательная статистика, а для обработки качественных данных - контент-анализ.

Результаты: В настоящем исследовании обнаружено, что птицеводческие фермы в кластерах едва ли имеют лучшие экономические показатели чем те, которые работают вне ППК. Многие фермеры в ППК считают, что те представляют преимущество для расширения масштаба работы по птицеводству и улучшению семейного дохода, но их меньше интересует - и у них ограничены возможности для этого - повышение биобезопасности и взаимодействие с окружающей средой. Нами замерялся уровень биобезопасности ферм в ППК посредством вопросника из 14 пунктов и было обнаружено, что биобезопасность в общем случае является очень низкой по всем местам выборки. Повышенная нагрузка в виде мух, комаров, крыс и запахов внутри и вокруг ППК не только загрязняет окружающую среду, но также вызывает социальные конфликты в окружающих сообществах.

Вывод: На основании настоящего исследования можно заключить, что птицеводческие кластеры, руководствующиеся, в основном, экономическими целями, едва ли являются лучшей моделью для контроля за инфекционными заболеваниями. Было обнаружено, что уровень биобезопасности ППК является низким. С учетом интенсивности птицеводческих работ в ППК (фермы плотно упакованы в кластеры) и близкого нахождения к жилым зонам некоторых ППК риск распространения инфекционных заболеваний фактически возрастает. Хорошее управление и коллективные действия по реализации мер по биобезопасности являются ключевыми для того, чтобы малые производители в ППК могли справится с обычными проблемами и руководствовались практиками животноводства на основе здоровья.

Translated from English version into Russian by Alexander Somin, through

TRANSLATORS
WITHOUT BORDERS

Evaluación ecoepidemiológica de granjas avícolas (PPC) para la mejora de la subsistencia y de la bioseguridad de los pequeños productores en Asia

Libin Wang, Edi Basuno, Tuan Nguyen, Worapol Aengwanich, Nyak Ilham, Xiaoyun Li

Resumen

Antecedentes: Los programas para granjas avícolas (PPC) son unas estrategias clave utilizadas en muchos países asiáticos para involucrar a los pequeños avicultores comerciales en grandes cadenas de producción y para controlar enfermedades infecciosas de las aves. El presente estudio ha evaluado las múltiples repercusiones de los PPC mediante un enfoque transdisciplinar en cuatro países asiáticos y ha sacado las conclusiones para que los pequeños productores mejoren sus medios de vida y reduzcan el riesgo de propagación de enfermedades infecciosas en el sector avícola.

Métodos: Se ha utilizado el método cuantitativo tanto como el cualitativo en la recopilación de datos. Dicho método ha constado de: cuestionarios formales estructurados a domicilio, medición de los niveles de bioseguridad de las granjas avícolas una tabla de clasificación y entrevistas con declarantes clave. Se ha utilizado la estadística descriptiva para procesar los datos cuantitativos, y el análisis de contenidos para el proceso de datos cualitativos.

Resultados: La investigación ha descubierto que las granjas avícolas que forman parte de un grupo no necesariamente tienen mejores resultados económicos que las que están fuera de los PPC. Muchos granjeros en PPC solo los consideran una ventaja porque aumentan número de sus operaciones y mejoran sus ingresos familiares, además les quitan preocupaciones en cuanto a –ya que tienen capacidad limitada para ello– mejorar la bioseguridad y la gestión medioambiental. Se ha medido el nivel de bioseguridad de las granjas en PPC mediante una lista de comprobación de 14 elementos y se ha hallado que por lo general el nivel es muy bajo en todas las ubicaciones de la muestra. El aumento de moscas, mosquitos, ratas y olores dentro y en los alrededores de los PPC no solo contamina el ambiente, sino que provoca conflictos sociales con las comunidades circundantes.

Conclusión: La presente investigación concluye que una agrupación avícola, principalmente promovida por objetivos económicos, no es necesariamente el mejor modelo de control de enfermedades infecciosas. Se ha hallado que el nivel de bioseguridad de los PPC es bajo. Dada la intensidad de la producción avícola en los PPC (las granjas están densamente apiñadas en grupos) y la proximidad de algunos de ellos a zonas residenciales, el riesgo de propagación de enfermedades infecciosas de hecho, aumenta. Una buena gestión y la acción colectiva para la ejecución de medidas de bioseguridad son clave para que los pequeños productores de los PPC aborden los problemas comunes y apliquen prácticas sanitarias en la manipulación de animales.

Translated from English version into Spanish by Raquel Bentué, through

TRANSLATORS
WITHOUT BORDERS
